# Clozapine Induces an Acute Proinflammatory Response That Is Attenuated by Inhibition of Inflammasome Signaling: Implications for Idiosyncratic Drug-Induced Agranulocytosis

**DOI:** 10.1093/toxsci/kfab154

**Published:** 2021-12-22

**Authors:** Samantha Christine Sernoskie, Alexandra R Lobach, Ryuji Kato, Alison Jee, Joseph Kyle Weston, Jack Uetrecht

**Affiliations:** 1 Department of Pharmaceutical Sciences, Faculty of Pharmacy, University of Toronto, Toronto, ON M5S 3M2, Canada; 2 Department of Cardiovascular Pharmacotherapy and Toxicology, Faculty of Pharmacy, Osaka Medical and Pharmaceutical University, Osaka 569-1094, Japan; 3 Department of Pharmacology and Toxicology, Temerty Faculty of Medicine, University of Toronto, Toronto, ON M5S 1A8, Canada

**Keywords:** clozapine, inflammation, inflammasome activation, idiosyncratic drug-induced agranulocytosis, caspase 1, innate immunity

## Abstract

Although clozapine is a highly efficacious schizophrenia treatment, it is under-prescribed due to the risk of idiosyncratic drug-induced agranulocytosis (IDIAG). Clinical data indicate that most patients starting clozapine experience a transient immune response early in treatment and a similar response has been observed in clozapine-treated rats, but the mechanism by which clozapine triggers this transient inflammation remains unclear. Therefore, the aim of this study was to characterize the role of inflammasome activation during the early immune response to clozapine using *in vitro* and *in vivo* models. In both differentiated and nondifferentiated human monocytic THP-1 cells, clozapine, but not its structural analogues fluperlapine and olanzapine, caused inflammasome-dependent caspase-1 activation and IL-1β release that was inhibited using the caspase-1 inhibitor yVAD-cmk. In Sprague Dawley rats, a single dose of clozapine caused an increase in circulating neutrophils and a decrease in lymphocytes within hours of drug administration along with transient spikes in the proinflammatory mediators IL-1β, CXCL1, and TNF-α in the blood, spleen, and bone marrow. Blockade of inflammasome signaling using the caspase-1 inhibitor VX-765 or the IL-1 receptor antagonist anakinra attenuated this inflammatory response. These data indicate that caspase-1-dependent IL-1β production is fundamental for the induction of the early immune response to clozapine and, furthermore, support the general hypothesis that inflammasome activation is a common mechanism by which drugs associated with the risk of idiosyncratic reactions trigger early immune system activation. Ultimately, inhibition of inflammasome signaling may reduce the risk of IDIAG, enabling safer, more frequent use of clozapine in patients.

Impact Statement These data indicate that caspase-1-dependent IL-1β production is fundamental for the induction of the early immune response to clozapine and, furthermore, support the general hypothesis that inflammasome activation is a common mechanism by which drugs associated with the risk of idiosyncratic reactions trigger early immune system activation. Ultimately, inhibition of inflammasome signaling may reduce the risk of idiosyncratic drug-induced agranulocytosis (IDIAG), enabling safer, more frequent use of clozapine in patients.

Clozapine (8-chloro-11-(4-methylpiperazin-1-yl)-5H-dibenzo[b, e][1,4]diazepine) has unparalleled efficacy in the management of schizophrenia (Numata *et al.*, 2018) but its use is precluded by the risk of IDIAG (Bogers *et al.*, 2016; Li *et al.*, 2020), a precipitous drop in circulating neutrophils that results in increased susceptibility to potentially fatal infections. Although the mechanistic basis of IDIAG has been the focus of decades of research (de la Chapelle *et al.*, 1977), the events preceding reaction onset remain poorly defined (Mijovic and MacCabe, 2020). Clozapine-induced IDIAG is most likely initiated by formation of reactive metabolites. Previous studies have demonstrated that a nitrenium ion is generated by myeloperoxidase-derived oxidants in neutrophils which, in turn, covalently binds to neutrophil proteins (Gardner *et al.*, 1998a, 2005; Liu and Uetrecht, 1995; Lobach and Uetrecht, 2014a) (Figure  1). Neoantigen formation alone, however, appears insufficient to cause this reaction, because irreversible binding of clozapine to patients’ neutrophils has been detected following chronic exposure in cases where patients do not develop agranulocytosis (Gardner *et al.*, 1998a).

**Figure 1.**
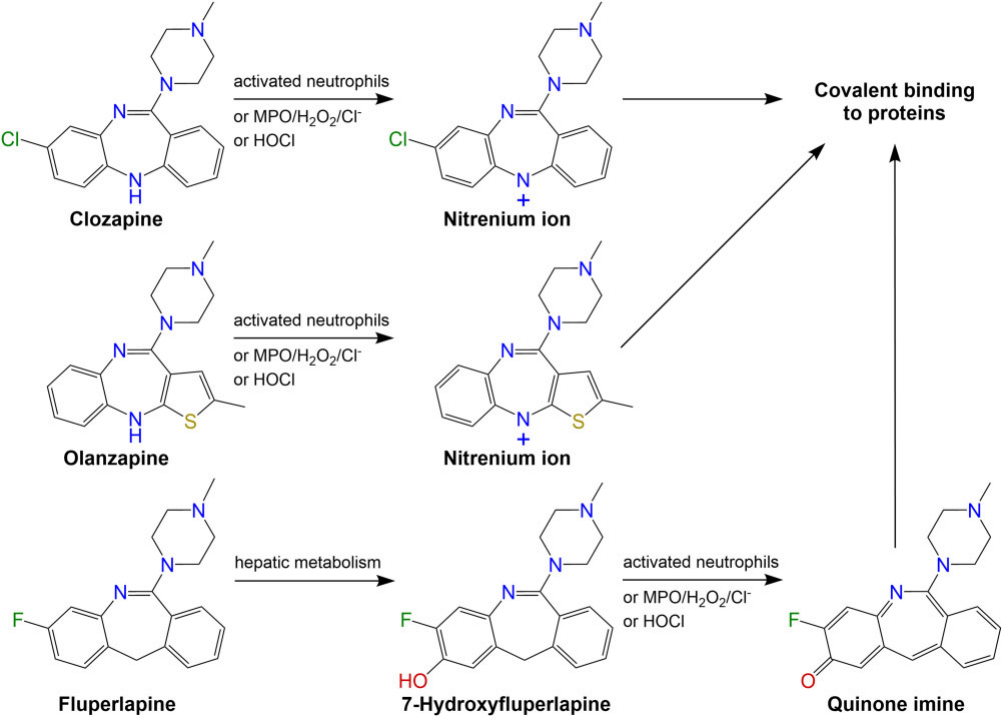
Proposed bioactivation pathways of clozapine, olanzapine, and fluperlapine. Clozapine and olanzapine are directly metabolized by activated neutrophils; oxidant systems containing myeloperoxidase (MPO), hydrogen peroxide (H_2_O_2_), and chloride; or hypochlorous acid, leading to reactive nitrenium ion formation and subsequent covalent binding to endogenous proteins ([Bibr kfab154-B12][Bibr kfab154-B13]; Liu and Uetrecht, 1995). Prior to formation of a reactive quinone immune species that can bind to proteins, fluperlapine must first be metabolized to 7-hydroxyfluperlapine, which can then be bioactivated by activated neutrophils or oxidant systems (Lai *et al.*, 2000).

Mandatory hematological monitoring has demonstrated that most patients develop a transient innate immune response within the first weeks of clozapine treatment, evidenced by paradoxical neutrophilia and increased levels of circulating proinflammatory mediators, including C-reactive protein (CRP), interleukin (IL)-6, and tumor necrosis factor (TNF)-α (Blackman *et al.*, 2021; Sernoskie *et al.*, 2021). Previous investigations have demonstrated that clozapine induces a similar acute increase in circulating neutrophils in rats (Lobach and Uetrecht, 2014b; Ng *et al.*, 2014; Wasti *et al.*, 2006) and rabbits (Iverson *et al.*, 2010). Notably, our group observed alterations in neutrophil kinetics over the first 10 days of daily clozapine administration in female Sprague Dawley rats, which was characteristic of bone marrow stimulation observed with increased G-CSF levels and consistent with the innate immune response that occurs early during clinical treatment in patients (Pollmächer *et al.*, 1997; Schuld *et al.*, 2000). Thus, this innate response is likely another necessary but, alone, insufficient step in the progression to agranulocytosis, which is believed to be an adaptive immune-mediated reaction associated with specific human leukocyte antigen (HLA) haplotypes (Chowdhury *et al.*, 2011; Legge and Walters, 2019). Specifically, mechanistic studies have demonstrated that an adaptive immune response mediated by CD4^+^ T cells is generated in response to the interaction of clozapine with HLA-DR molecules (Ogese *et al.*, 2020); a response which must be preceded by innate immune activation.

One mechanism by which covalent binding of clozapine may trigger an innate response is through inflammasome activation. Inflammasomes are a class of multimeric protein complexes that, when activated, lead to the maturation of caspase-1, the cleavage and release of IL-1β and IL-18, and the induction of a proinflammatory response (Schroder and Tschopp, 2010). Several inflammasomes are activated in response to the detection of nonpathogenic stimuli or damage-associated molecular patterns (DAMPs), which could be released following cellular damage induced by clozapine covalent binding. This potential mechanism of clozapine neoantigen-induced immune activation parallels the response to contact allergens, which are also reactive compounds that modify endogenous proteins to induce an immune response (Martin, 2015). Notably, it has been shown that rodents deficient in inflammasome subunits have an impaired response to contact sensitizers (Watanabe *et al.*, 2007). Additionally, a role for the NLRP3 inflammasome has been demonstrated in adverse reactions to abacavir (Toksoy *et al.*, 2017) and acetaminophen (Imaeda *et al.*, 2009), underlying the expanding importance of innate immune signaling in adverse drug reactions.

Previously, we demonstrated that several drugs associated with the risk of various idiosyncratic drug reactions (IDRs) directly (Kato and Uetrecht, 2017; Weston and Uetrecht, 2014) or indirectly (Kato and Uetrecht 2017; Kato *et al.*, 2019, 2020) activate inflammasomes in phorbol 13-myristate 12-acetate (PMA)-differentiated human monocytic THP-1 cells. PMA-differentiated THP-1 macrophages exhibit increased gene expression of IL-1β in comparison with undifferentiated monocytes, providing a sensitive platform to detect inflammasome activation by IDR-associated drugs. However, a role for inflammasome activation by clozapine *in vitro* and, more broadly, with IDR-associated drugs *in vivo* remains to be defined.

Thus, we hypothesized that inflammasome activation and IL-1β release occur following exposure to clozapine, driving a rapid and transient proinflammatory immune response. The objectives of this study were to (1) characterize induction of the early immune response to clozapine in comparison with the structurally related drugs olanzapine (2-methyl-4-(4-methyl-1-piperazinyl)-10H-thieno[2,3-b][1,5]benzodiazepine) and fluperlapine (3-fluoro-6-(4-methylpiperazin-1-yl)-11H-dibenzo[b, e]azepine) (Figure  1) and (2) demonstrate the requisite of inflammasome signaling in this proinflammatory response.

**Figure 2.**
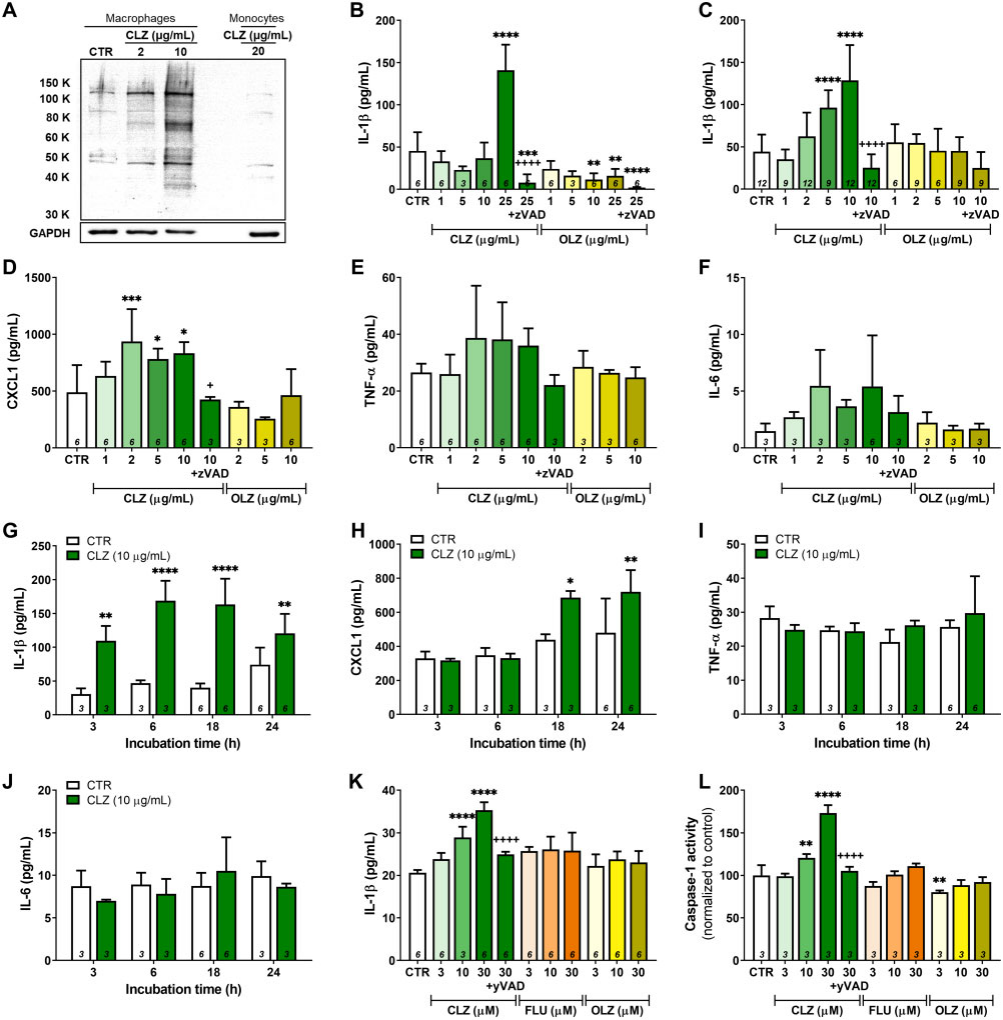
Clozapine directly activates inflammasomes in THP-1 cells. A, Immunoblot analysis to detect covalent binding of clozapine to THP-1 cells. Macrophages were incubated with 0.25% DMSO (CTR), 2, or 10 μg/ml of clozapine for 24 h and monocytes were incubated with 20 μg/ml of clozapine for 24 h. Extracted cell proteins (10 μg) were run on an 8% polyacrylamide gel, transferred to 0.2 μm nitrocellulose membranes, and incubated with anticlozapine antiserum (1:1000 dilution), prior to chemiluminescent detection of covalent binding. Membranes were then stripped and reprobed with the loading control glyceraldehyde 3-phosphate dehydrogenase (GAPDH; 1:20000 dilution). Membranes were imaged using a FluoroChem Alpha Innotech imager with AlphaEase FC software, version 6.0.0 (ProteinSimple, Santa Clara, California). B and C, Supernatant concentrations of IL-1β in THP-1 monocytes (B) or in THP-1-derived macrophages (C) following 24 h of incubation with clozapine (1–25 μg/ml), olanzapine (1–25 μg/ml), clozapine (25 μg/ml) with the pan-caspase inhibitor zVAD-fmk (10 μg/ml), or olanzapine (25 μg/ml) with zVAD-fmk (10 μg/ml) (1-way ANOVA with Holm-Sidak’s multiple comparison test). D–F, Supernatant concentrations of CXCL1 (D), TNF-α (E), or IL-6 (F) in THP-1-derived macrophages following 24 h of incubation with clozapine (1–10 μg/ml), olanzapine (10 μg/ml), or clozapine (10 μg/ml) with the pan-caspase inhibitor zVAD-fmk (10 μg/ml) (1-way ANOVA with Holm-Sidak’s multiple comparison test). G–J, Supernatant concentrations of IL-1β (G), CXCL1 (H), TNF-α (I), or IL-6 (J) in THP-1-derived macrophages up to 24 h following incubation with clozapine (10 μg/ml) (1-way ANOVA with Holm-Sidak’s multiple comparison test). K and L, Supernatant concentrations of IL-1β (K) or normalized caspase-1 activity (L) in THP-1-derived macrophages following 24 h of incubation with clozapine (1–10 μg/ml), fluperlapine (1–10 μg/ml [3–30 μM]), olanzapine (1–10 μg/ml [3–30 μM]), or clozapine (10 μg/ml [30 μM]) with the caspase-1 inhibitor yVAD-cmk (0.54 μg/ml) (1-way ANOVA with Holm-Sidak’s multiple comparison test). Inflammatory mediators were quantified using commercially available ELISAs and caspase-1 activity was quantified using a Promega Caspase-Glo 1 Inflammasome Assay. CTR, control; CLZ, clozapine; OLZ, olanzapine; FLU, fluperlapine; *, *p* < .05; **, *p* < .01; ***, *p* < .001; ****, *p* < .0001 (vs CTR); ^+^, *p* < .05; ^++++^, *p* < .0001 (vs highest concentration of drug without inhibitor).

## MATERIALS AND METHODS

### Reagents

Clozapine was donated by Novartis Pharmaceuticals Inc. (Dorval, QC) and olanzapine was purchased from Toronto Research Chemicals Inc. (Toronto, ON). Fluperlapine and the selective caspase-1 inhibitor VX-765 ((S)-1-((S)-2-{[1-(4-Amino-3-chloro-phenyl)-methanoyl]-amino}-3,3-dimethyl-butanoyl)-pyrrolidine-2-carboxylic acid ((2R, 3S)-2-ethoxy-5-oxo-tetrahydro-furan-3-yl)-amide) were purchased from MedChemExpress (Princeton, New Jersey) and provided by LB Pharmaceuticals Inc. (New York, New York). The pan-caspase inhibitor zVAD-fmk (benzyloxycarbonyl-Val-Ala-Asp (OMe) fluoromethylketone) was acquired from InvivoGen (San Diego, California), the caspase-1 inhibitor yVAD-cmk (acetyl-tyrosyl-valyl-alanyl-aspartyl-chloromethylketone) was purchased from Promega Corporation (Madison, Wisconsin), and the clinical formulation of the IL-1 receptor antagonist anakinra (Amgen, Thousand Oaks, California) was obtained from a local pharmacy. Lipopolysaccharides (LPS) and PMA were purchased from Sigma-Aldrich (St Louis, Missouri). All other reagents were commercially obtained.

### Cell culture

THP-1 monocytes (4 × 10^5^ cells/ml; American Type Culture Collection [ATCC], Manassas, Virginia) were cultured in ATCC high glucose RPMI-1640 media, supplemented with 10% fetal bovine serum and 2-mercaptoethanol. Following a 3-h stimulation with LPS (1 μg/ml), monocytes were washed with PBS (Ca^2+/^Mg^2+^-free) and resuspended in media lacking 2-mercaptoethanol. Cells were incubated for 24 h at 37°C, 5% CO_2_, with clozapine or olanzapine (1–25 μg/ml), dissolved in 0.25% DMSO. The pan-caspase inhibitor zVAD-fmk (10 μg/ml) was coincubated with both drugs. Cell culture supernatants were collected for analysis. To detect clozapine covalent binding, THP-1 cells were lysed with 1× cell lysis buffer (Cell Signaling Technologies, Pickering, ON) with 1× Halt protease inhibitor cocktail (Pierce Biotechnology, Rockford, Illinois). Western blots were carried out using an anticlozapine antibody developed in-house, as described previously (Lobach and Uetrecht, 2014a).

In some experiments, THP-1 monocytes were first differentiated to macrophages with 3 days of PMA (50 ng/ml) stimulation at 37°C, 5% CO_2_. Cells were washed, resuspended in media, and incubated for 24 h prior to incubation in fresh media for up to 24 h with clozapine, fluperlapine, or olanzapine (1–10 μg/ml; 3–30 μM) dissolved in 0.25% DMSO. Clozapine and olanzapine were coincubated with zVAD-fmk (10 μg/ml); clozapine was also coincubated with the caspase-1 inhibitor yVAD-cmk (0.54 μg/ml). Cells were incubated at 37°C for 6–24 h and supernatants were collected for analysis.

### Animal treatment and drug administration

Female Sprague Dawley rats (200–250 g) were obtained from Charles River (St Constant, QC) and double or triple housed with a 12/12-h light/dark cycle at 22°C. Animals were acclimatized for >1 week and were provided access to rodent chow (Harlen Teklad, Madison, Wisconsin) and water *ad libitum*. All animal protocols were approved by the University of Toronto Animal Care Committee. Clozapine and fluperlapine were dissolved in 1 M hydrochloric acid, diluted in saline, and adjusted to pH 6 with 1 M sodium hydroxide. Clozapine (30 mg/kg) or vehicle (saline) was administered between 8 and 9 am via intraperitoneal (IP) injection. To analyze the effect of inflammasome modulation on immune outcomes, rats either received a single predose of VX-765 (50 mg/kg) or vehicle (10% dimethyl sulfoxide [DMSO] in phosphate-buffered saline [PBS]) via gavage 1 h prior to a single clozapine dose. An equimolar dose of fluperlapine (28.4 mg/kg, IP) was also assessed. In a separate study, clozapine-treated rats received anakinra (50 mg/kg) or vehicle (PBS) via subcutaneous (SC) injection as predoses administered 24- and 1-h pre-clozapine.

### Tissue collection

Whole blood was collected for differential blood counts using a VetScan HM5 (Union City, California) or manual counts were conducted using Turk’s blood diluting fluid (Ricca Chemical Company, Arlington, Texas). Leukocyte differentials were calculated by classifying >100 leukocytes on blood smears stained with Giemsa-Wright-like stain (Cambridge Diagnostic Products, Fort Lauderdale, Florida). In some studies, spleen and bone marrow were collected and homogenized using ice-cold Dounce homogenizers and 1× radioimmunoprecipitation assay buffer (RIPA) buffer (Abcam, Toronto, ON), supplemented with protease and phosphatase inhibitor cocktail (Abcam). For measurement of capsase-1 activity, homogenates were prepared without protease inhibitors, per the kit’s instructions.

### Inflammatory mediator measurement

The following ELISA kits were used for rat tissue: IL-1β, TNF-α, chemokine ligand 1 (CXCL1), IL-6, and α-1-acid glycoprotein (α-1-AGP) (R&D Systems, Minneapolis, Minnesota). The following ELISA kits were used for THP-1 cell culture supernatants: IL-1β (Life Technologies Inc., Burlington, ON) and TNF-α, CXCL1, and IL-6 (R&D Systems, Minneapolis, Minnesota).

### Caspase-1 activity measurement

Caspase-1 activity in rat tissue was measured using a fluorometric Caspase-1 Assay Kit according to the kit instructions (Abcam, Toronto, ON). Recombinant human caspase-1 (0.125–2 U; Sigma-Aldrich, Oakville, ON) was used as a positive control, and treated sample activity was normalized to control. Caspase-1 activity in differentiated THP-1 cells was measured using a Caspase-Glo 1 Inflammasome Assay (Promega Corporation, Madison, Wisconsin), as described previously (Kato *et al.*, 2021).

### Statistical analysis

Results are expressed as means ± SD, analyzed using GraphPad Prism software version 8.4.3 (GraphPad, San Diego, California). Differences between groups were assessed using Student’s *t* test or 1- or 2-way ANOVA with the Holm-Sidak’s multiple comparison test. An adjusted *p* value < .05 was considered statistically significant.

## RESULTS

### Inflammasome Activation Is Increased With Clozapine *In Vitro*

To investigate whether clozapine can activate inflammasomes *in vitro*, similar to other drugs associated with IDRs (Imano *et al.*, 2021; Kato and Uetrecht 2017; Kato *et al.*, 2019, 2020, 2021; Weston and Uetrecht, 2014), THP-1 monocytes, and PMA-differentiated macrophages were treated with clozapine and the structural analogues olanzapine and fluperlapine. First, to confirm that a clozapine reactive metabolite was generated, covalent binding to THP-1 cells was assessed by Western blotting. Although binding of clozapine (20 μg/ml) to THP-1 monocytes did not appear to be greater than the negative control, a concentration-dependent increase in covalent binding was observed in differentiated THP-1 macrophages (Figure  2A).

Having verified that THP-1 cells bioactivate clozapine, production of IL-1β, and other proinflammatory cytokines were assessed. Incubation of THP-1 monocytes or macrophages with clozapine (25 μg/ml and 3–10 μg/ml, respectively) for 24 h increased IL-1β production (*p* < .0001) whereas the negative controls olanzapine or fluperlapine did not (Figs.  2B, C, and K). THP-1 macrophages were also found to release the neutrophil chemokine CXCL1 after 24-h incubation with 2–10 μg/ml clozapine (*p *< .001, *p* < .05, and *p* < .05) (Figure  2D). In a 24-h time course of THP-1 macrophages exposed to 10 μg/ml clozapine, increased levels of IL-1β were detected from 3 to 24 h (*p* < .01) (Figure  2G), and CXCL1 release increased at 18 and 24 h (*p *< .05 and *p* < .01, respectively) (Figure  2H). No statistically significant changes were observed with TNF-α or IL-6 in the 24-h and the time course analyses with THP-1 macrophages (Figs.  2E, F, I, and J).

Given the marked increases in proinflammatory mediator generation with clozapine, the last approach was to inhibit inflammasome activation. Production of IL-1β in clozapine-treated THP-1 monocytes and macrophages was completely blocked by the caspase inhibitor zVAD-fmk (*p* < .0001) (Figs.  2B and 2C), confirming that IL-1β was generated in a caspase-dependent manner. Similarly, CXCL1 release in THP-macrophages was blocked by zVAD-fmk (*p* < .05) (Figure  2D). Caspase-1 activity was increased in clozapine-treated THP-1 macrophages (3 and 10 μg/ml; *p* < .01 and *p* < .0001, respectively) and coincubation with clozapine and the caspase-1 inhibitor yVAD-cmk decreased caspase-1 activity and prevented IL-1β release (*p* < .0001) (Figs.  2K and 2L). Together, these findings support the role of caspase-1-dependent inflammasome activation in the mechanism of the early proinflammatory response induced by clozapine.

### Acute Immune Cell Changes With Clozapine *In Vivo*

**Figure 3.**
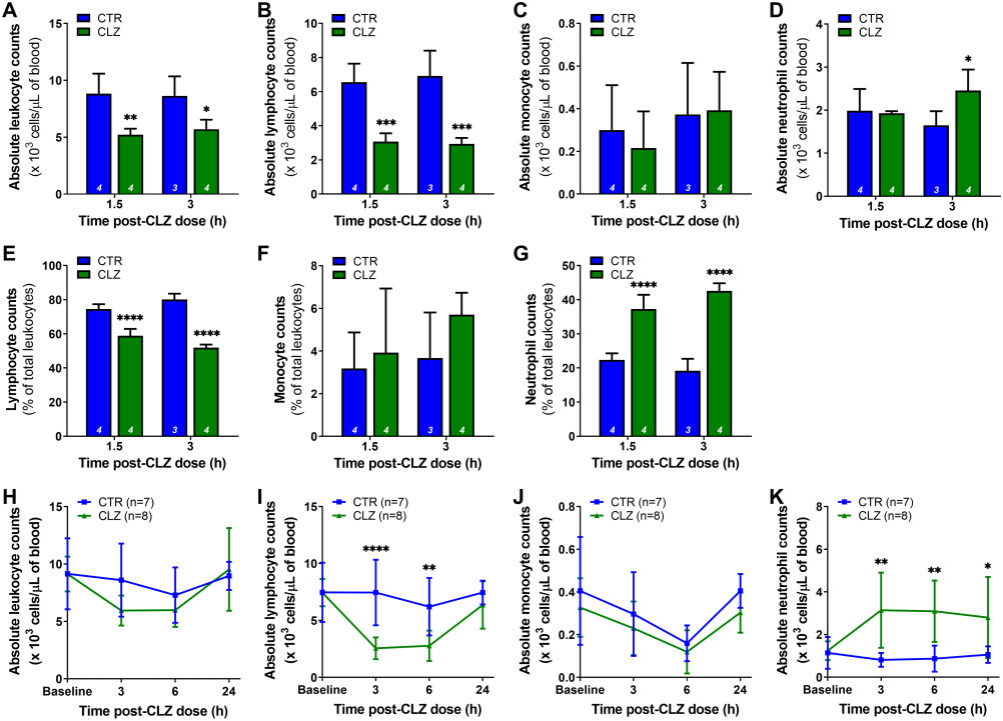
Clozapine rapidly triggers an increase in blood neutrophil counts and a decrease in lymphocyte counts in Sprague Dawley rats. A–D, Absolute leukocyte (A), lymphocyte (B), monocyte (C), and neutrophil (D) counts, presented as 10^3^ cells/μl of blood, at 1.5 and 3 h following a single dose of clozapine (30 mg/kg, IP) in female Sprague Dawley rats (1-way ANOVA with Holm-Sidak’s multiple comparison test). E–G, Corresponding lymphocyte (E), monocyte (F), and neutrophil (G) counts, presented as a percentage of total leukocytes, at 1.5 and 3 h post clozapine (1-way ANOVA with Holm-Sidak’s multiple comparison test). Differential blood counts were determined using a VetScan HM5. H–K, Absolute leukocyte (H), lymphocyte (I), monocyte (J), and neutrophil (K) counts, presented as 10^3^ cells/μl of blood, over a 24-h period post-clozapine (2-way ANOVA with Holm-Sidak’s multiple comparison test). Differential blood counts were determined manually. CTR, control; CLZ, clozapine; *, *p* < .05; **, *p* < .01; ***, *p* < .001; ****, *p* < .0001.

Neutrophil increases have been demonstrated in patients beginning clozapine treatment (Blackman *et al.*, 2021; Sernoskie *et al.*, 2021) and in our rat model (Abdel-Wahab and Metwally, 2014; Lobach and Uetrecht, 2014b; Ng *et al.*, 2014; Wasti *et al.*, 2006). To examine the induction of this immune response in the rat model, changes in leukocyte populations were evaluated over 24 h following a single clozapine dose (30 mg/kg). Using liquid chromatography-mass spectrometry (LC-MS), it was previously demonstrated that this dose yields serum drug levels that approximate the average therapeutic range reported in patients (Lobach and Uetrecht, 2014b). A significant reduction in total circulating leukocytes at 1.5 h (*p* < .01) (Figure  3A) was observed that was driven by a decrease in lymphocytes, detected both as an absolute cell count (*p* < .001) and as a percentage of leukocytes (*p* < .0001) (Figs.  3B and 3E). Decreased lymphocyte levels were sustained at 3 h and accompanied by an increase in circulating neutrophils (absolute counts: *p* < .05; percentage: *p* < .0001) (Figs.  2D and 2G). Monocyte counts were unchanged (Figs.  3C and 3F). Similar findings were observed at 3 h using manual blood counts, with changes beginning to resolve by 24 h (Figs.  3H–K).

### Acute Inflammatory Mediator Changes With Clozapine *In Vivo*

**Figure 4.**
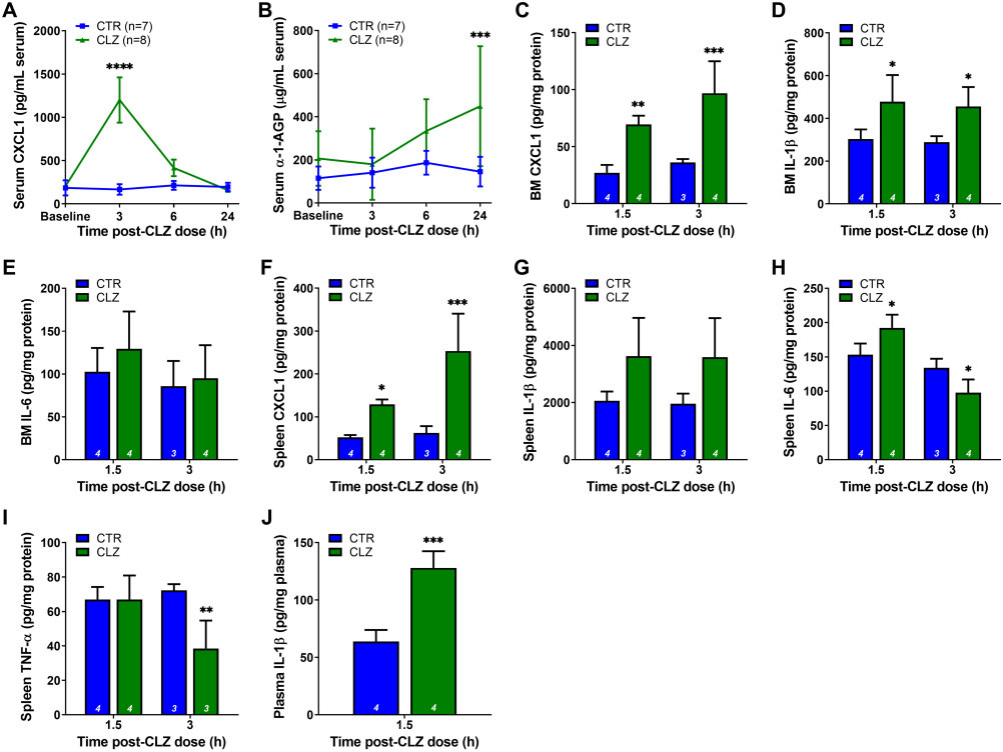
Clozapine causes an acute increase in blood- and tissue-derived proinflammatory mediators. A and B, Serum concentrations of CXCL1 (A) and α-1-AGP (B) over a 24-h period following a single dose of clozapine (30 mg/kg, IP) in female Sprague Dawley rats (ANOVA with Holm-Sidak’s multiple comparison test). C–E, Bone marrow concentrations of CXCL1 (C), IL-1β (D), and IL-6 (E) at 1.5 and 3 h post clozapine (ANOVA with Holm-Sidak’s multiple comparison test). F–I, Spleen concentrations of CXCL1 (F), IL-1β (G), IL-6 (H), and TNF-α (I) at 1.5 and 3 h post clozapine (ANOVA with Holm-Sidak’s multiple comparison test). (J) Plasma concentrations of IL-1β at 1.5 h post-clozapine (Student’s *t* test). Inflammatory mediators were quantified using commercially available ELISAs. CTR, control; CLZ, clozapine; BM, bone marrow; *, *p* < .05; **, *p* < .01; ***, *p* < .001; ****, *p* < .0001.

In addition to circulating immune cell changes, patients starting clozapine treatment exhibit increases in serum levels of various proinflammatory markers, including CRP, IL-6, TNF-α, and G-CSF (Löffler *et al.*, 2010; Pollmacher *et al.*, 1996, 1997). G-CSF, a known regulator of neutrophil proliferation and activation, also transiently increases in our clozapine rat model from 3 to 6 h following a single dose but not with repeated daily treatment (Lobach and Uetrecht, 2014b). To further examine the proinflammatory mediators that could trigger rapid changes in circulating leukocyte populations in the rat model, cytokine levels were investigated in the blood, bone marrow, and spleen over 24 h following a single clozapine dose (30 mg/kg). Serum levels of CXCL1 increased at 3 h post dose (*p* < .0001) and returned to control level by 24 h (Figure  4A), whereas α-1-AGP (a rat acute phase inflammatory protein comparable with human CRP) was significantly elevated at 24 h (*p* < .001) (Figure  4B). Serum TNF-α, IL-1β, and IL-6 were undetectable at the 3, 6, and 24-h timepoints, whereas in a shorter time course, IL-1β was found to spike in the plasma at 1.5 h (*p* < .0001) (Figure  4J). In the bone marrow, CXCL1 and IL-1β were significantly elevated at 1.5 h (*p* < .01 and *p* < .05, respectively) and 3 h (*p* < .001 and *p* < .05, respectively), whereas TNF-α was undetectable, and IL-6 was unchanged (Figs.  4C–E). In the spleen, CXCL1 and IL-6 were significantly elevated at 1.5 h (*p* < .05 and *p* < .01), and CXCL1 was further elevated at 3 h (*p* < .001), whereas IL-6 and TNF-α were significantly reduced at 3 h (both *p* < .05) (Figs.  4F, H, and I). Splenic levels of IL-1β trended toward an increase at both 1.5 and 3 h but did not reach statistical significance (*p* = .054 and *p* = .061, respectively) (Figure  4G).

### Inflammasome Activation Is Necessary for the Immune Response to Clozapine *In Vivo*

**Figure 5.**
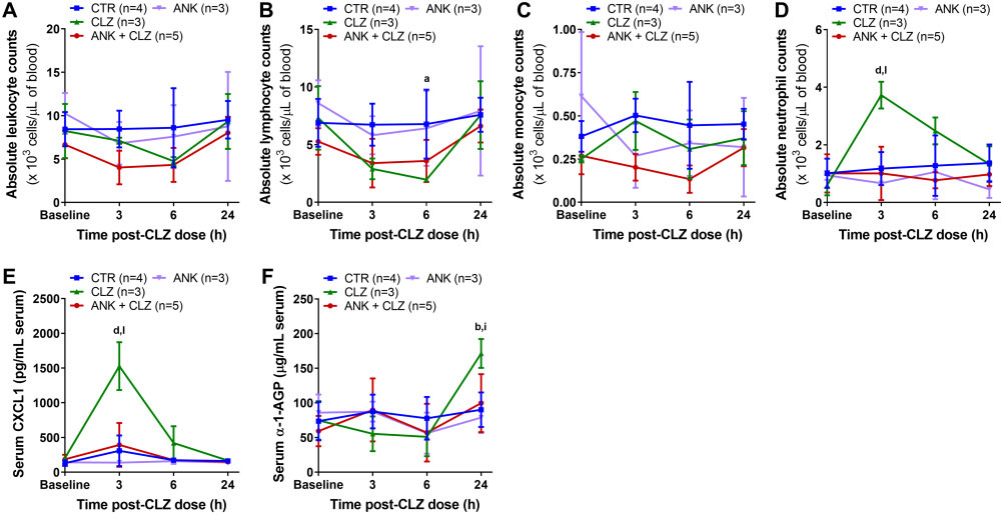
IL-1 receptor antagonism attenuates the proinflammatory response induced by clozapine. A–D, Absolute leukocyte (A), lymphocyte (B), monocyte (C), and neutrophil (D) counts, presented as 10^3^ cells/μl of blood, in female Sprague Dawley rats over 24 h following treatment with clozapine (30 mg/kg, IP), the IL-1 receptor antagonist anakinra (50 mg/kg, SC), or anakinra and clozapine (2-way ANOVA with Holm-Sidak’s multiple comparison test). Differential blood counts were determined manually. E and F, Serum concentrations of CXCL1 (E) and α-1-AGP (F) up to 24 h post-clozapine administration (2-way ANOVA with Holm-Sidak’s multiple comparison test). Inflammatory mediators were quantified using commercially available ELISAs. CTR, control; CLZ, clozapine; ANK, anakinra; ^a^, *p* < .05; ^b^, *p* < .01; ^d^, *p* < .0001 (CTR vs CLZ); ^i^, *p* < .05; ^l^, *p* < .0001 (CLZ vs ANK + CLZ).

To determine the role of inflammasome activation during induction of the immune response to clozapine in our rat model, inhibitors of two separate signaling components of inflammasomes were tested. First, rats were cotreated with the IL-1 receptor antagonist anakinra. Although there was a trend toward a decrease in the total leukocyte and lymphocyte counts at 3 and 6 h following cotreatment of anakinra and clozapine, these changes were not statistically different from controls (Figs.  5A and 5B). IL-1 receptor antagonism did significantly supress clozapine-induced neutrophil count increases at 3 and 6 h (*p* < .0001 and *p* < .01, respectively), without impacting monocyte counts (Figs.  5C and 5D). Anakinra cotreatment was also found to markedly attenuate the clozapine-mediated increases in CXCL1 at 3 h (*p* < .0001) and α-1-AGP at 24 h (*p* < .05) (Figs.  5E and 5F). Because CXCL1 was found to be generated in an inflammasome-dependent mechanism in THP-1 cells (Figure  2D) and by other groups (Tomalka *et al.*, 2011; Xu *et al.*, 2021), these data suggest that inflammasome activation is necessary for downstream amplification of the innate immune response to clozapine *in vivo*, as well. However, upon repeating this study using a different lot of anakinra, anakinra alone caused neutrophilia. Therefore, a second method of inflammasome signaling inhibition was explored.

**Figure 6.**
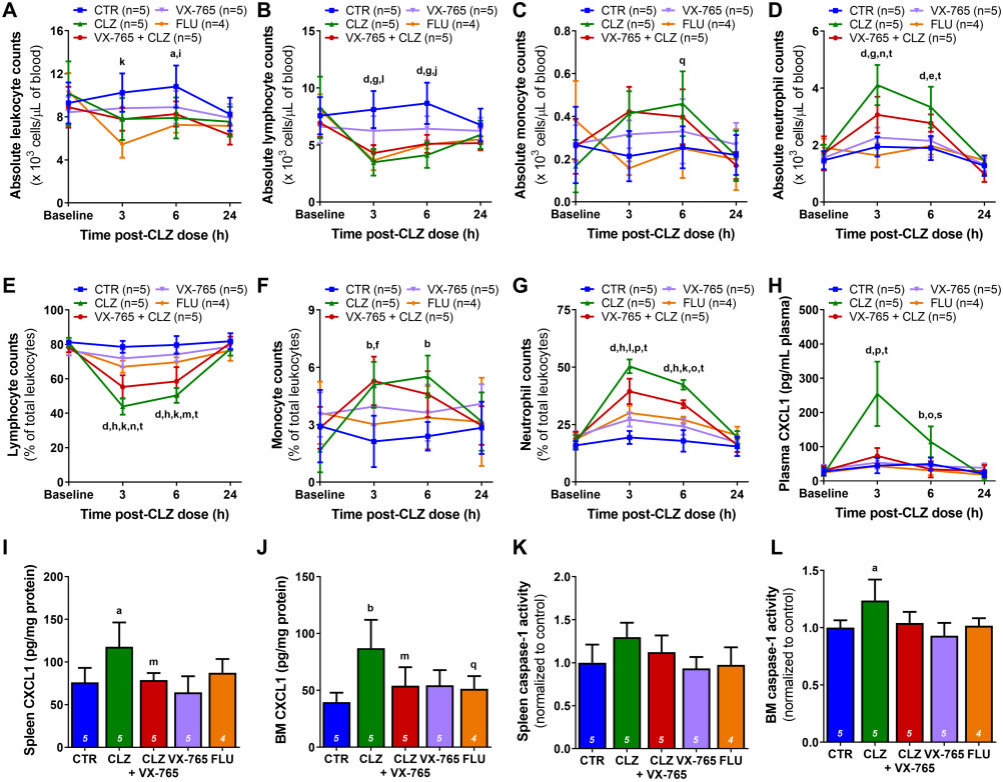
Caspase-1 inhibition attenuates the proinflammatory response induced by clozapine. A–D, Absolute leukocyte (A), lymphocyte (B), monocyte (C), and neutrophil (D) counts, presented as 10^3^ cells/μl of blood, in female Sprague Dawley rats over 24 h following treatment with vehicles (control), clozapine (30 mg/kg, IP), fluperlapine (28.4 mg/kg, IP), the caspase-1 inhibitor VX-765 (50 mg/kg, oral gavage), or VX-765 and clozapine (2-way ANOVA with Holm-Sidak’s multiple comparison test). E–G, Corresponding lymphocyte (E), monocyte (F), and neutrophil (G) counts, presented as a percentage of total leukocytes (2-way ANOVA with Holm-Sidak’s multiple comparison test). Differential blood counts were determined using a VetScan HM5. (H) Plasma concentrations of CXCL1 up to 24 h post-clozapine administration (2-way ANOVA with Holm-Sidak’s multiple comparison test). I and J Spleen (I) and bone marrow (J) concentrations of CXCL1 at 24 h post clozapine administration (1-way ANOVA with Holm-Sidak’s multiple comparison test). Inflammatory mediators were quantified using commercially available ELISAs. K and L Spleen (K) and bone marrow (L) caspase-1 activity at 24 h post clozapine administration, normalized to control values (1-way ANOVA with Holm-Sidak’s multiple comparison test). Caspase-1 activity was quantified using an Abcam fluorometric Caspase 1 Assay Kit. CTR, control; CLZ, clozapine; FLU, fluperlapine; BM, bone marrow; ^a^, *p* < .05; ^b^, *p* < .01; ^d^, *p* < .0001 (CTR vs CLZ); ^e^, *p* < .05; ^f^, *p* < .01; ^g^, *p* < .001; ^h^, *p* < .0001 (CTR vs VX-765 + CLZ); ^i^, *p* < .05; ^j^, *p* < .01; ^k^, *p* < .001; ^l^, *p* < .0001 (CTR vs FLU); ^m^, *p* < .05; ^n^, *p* < .01; ^o^, *p* < .001; ^p^, *p* < .0001 (CLZ vs VX-765 + CLZ); ^q^, *p* < .05; ^s^, *p* < .001; ^t^, *p* < .0001 (CLZ vs FLU).

Alternatively, rats were pretreated with the selective caspase-1 inhibitor VX-765 prior to clozapine administration. Inhibition of caspase-1 moderately dampened the clozapine-induced neutrophilia at 3 h (absolute counts: *p* < .01; percentage: *p* < .0001), without rescuing the decreased lymphocyte counts (Figs.  6A–G). Caspase-1 inhibition also reduced the elevated levels of CXCL1 observed with clozapine alone in the plasma at 3 h (*p* < .0001) and bone marrow at 24 h (*p* < 05), whereas caspase-1 activity levels were not significantly different (Figs.  6H, J, and L). Fluperlapine (28.4 mg/kg) was also tested here, and although fluperlapine caused a similar decrease in lymphocytes compared with clozapine (*p* < .0001), no increase in neutrophils or CXCL1 was detected, indicating that fluperlapine does not induce a similar innate immune response.

## DISCUSSION

IDIAG is believed to be an adaptive immune-mediated reaction associated with specific HLA haplotypes (Chowdhury *et al.*, 2011; Legge and Walters, 2019) and T cell receptors (Ogese *et al.*, 2020). However, induction of this adaptive response must be preceded by innate immune activation; an early response that does not appear to be idiosyncratic (Sernoskie *et al.*, 2021). Previously, we began characterizing the innate response to clozapine using a rat model (Lobach and Uetrecht, 2014b; Ng *et al.*, 2014). However, the mechanism responsible for induction of clozapine-induced neutrophilia remained unclear. The present work investigated the role of inflammasome signaling during the acute immune response to clozapine, providing compelling evidence that (1) caspase-1-mediated generation of IL-1β occurs in response to clozapine exposure *in vitro* and (2) blockade of inflammasome activation attenuates the proinflammatory response to clozapine *in vivo*. Increased circulating neutrophils and proinflammatory mediators (eg, TNF-α, IL-1β) in the blood, spleen, and bone marrow of rats were observed within hours of clozapine exposure, indicative of a rapid, highly coordinated multiorgan immune response. Linking back to our previous work, numerous cytokines released during inflammation have been found to upregulate proliferation and maturation of progenitor populations, causing increased granulopoiesis (Mitroulis *et al.*, 2020), and this has been observed in clozapine-treated patients (Røge *et al.*, 2012) and in our rat model (Lobach and Uetrecht, 2014b).

**Figure 7.**
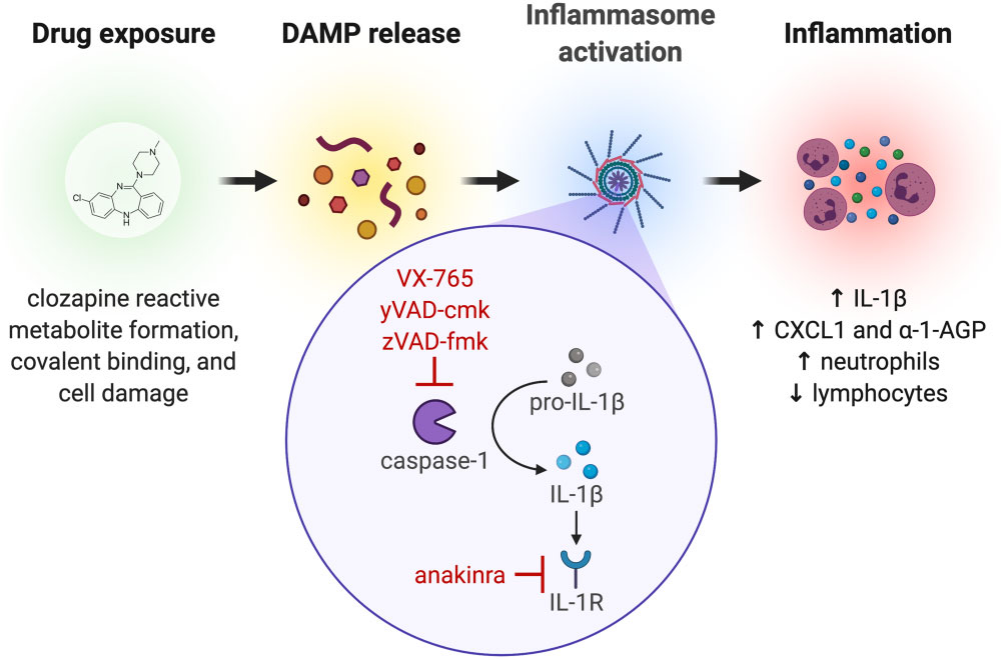
Proposed model of clozapine-induced inflammasome activation. Clozapine is bioactivated to a reactive metabolite that irreversibly binds to endogenous proteins, resulting in the generation of neoantigens (ie, haptens). If essential cellular proteins are haptenized, this causes cell stress, damage, or death that, in turn, triggers the release of DAMPs (including drug-modified proteins) and other proinflammatory stimuli from the injured cell. These signals drive the activation of local immune cells such as macrophages that can respond to DAMPs in several ways, including propagation of an inflammatory response via inflammasome activation. Inflammasome-dependent activation of caspase-1 increases the production of mature IL-1β from its inactive form. The released IL-1β induces a proinflammatory state through autocrine and paracrine signaling via the IL-1 receptor (IL-1R), leading to increased release of chemokines and proinflammatory cytokines (ie, CXCL1) and acute phase proteins (ie, α-1-AGP), as well as increased activation and mobilization of peripheral immune cells. VX-765, yVAD-cmk, and zVAD-fmk directly prevent clozapine-induced inflammasome activation through caspase-1 inhibition and anakinra prevents inflammasome signaling through antagonism of IL-1R, ultimately attenuating multiple components of the proinflammatory response to clozapine.

The current results define a novel role for inflammasome activation in response to clozapine treatment and, to our knowledge, demonstrate for the first time *in vivo* that inflammasome signaling is necessary for the innate immune response to an IDR-associated drug. To encapsulate these findings, we propose a model whereby cells, damaged by clozapine haptenization, release DAMPs that activate inflammasomes in myeloid cells, including macrophages and neutrophils (Figure  7). These activated cells produce inflammasome-dependent cytokines including IL-1β and CXCL1, driving a transient, proinflammatory immune response involving multiple organs and immune cell populations. Through inhibition of caspase-1 using VX-765, yVAD-cmk, or zVAD-fmk or antagonism of IL-1 receptor signaling using anakinra, clozapine-induced inflammasome activation is prevented and subsequent inflammation is attenuated. We postulate that this innate response is a necessary, but alone insufficient, step in the mechanism that, in some patients, precedes IDIAG: a reaction that appears to be mediated by the adaptive immune system (Johnston and Uetrecht, 2015). This innate response is required for activation of antigen presenting cells but, presumably, will not result in IDIAG without T cell activation and clonal expansion (Sernoskie *et al.*, 2021), necessitating HLA molecules that can present clozapine-modified peptides to cognate T cell receptors.

Several groups have reported clozapine-mediated hepatic (Jia *et al.*, 2014; Zlatkovic *et al.*, 2014), cardiac (Abdel-Wahab and Metwally, 2015; Mohammed *et al.*, 2020; Nikolic-Kokic *et al.*, 2018; Wang *et al.*, 2008), splenic (Abdelrahman *et al.*, 2014; Mohammed *et al.*, 2020), and other (Lobach and Uetrecht, 2014b; Ng *et al.*, 2014; Wasti *et al.*, 2006) immune changes but, aside from our group, these effects have been reported following several weeks, not hours, of clozapine administration (reviewed in Sernoskie *et al.* [2021]). The differences in inflammatory mediator changes observed in the spleen and bone marrow in the present study not only underscore the complexity of the immune response to clozapine, but the observed increases in IL-1β and other proinflammatory mediators support further investigation of organ-specific inflammasome activation in this response *in vivo*.

To our knowledge, only one other study has examined clozapine and inflammasome activation, but in a very different context (Giridharan *et al.*, 2020). In a model of poly (I: C)-induced inflammation using cultured primary microglial cells, clozapine was found to attenuate increased proinflammatory cytokines, NLRP3 mRNA expression, and caspase-1 activity compared with poly (I: C)-treated control cells. Cytokine changes were not observed with clozapine in the absence of poly (I: C), making it challenging to compare these results with the present study.

Investigations conducted by our group and collaborators have demonstrated that inflammasome activation occurs *in vitro* with numerous IDR-associated drugs and/or their metabolites but does not occur with structurally similar drugs that are not associated with IDRs (Imano *et al.*, 2021; Kato and Uetrecht 2017; Kato *et al.*, 2019, 2020, 2021; Weston and Uetrecht, 2014). Olanzapine and fluperlapine have structures similar to clozapine, where the former forms an analogous reactive nitrenium ion but is not associated with IDIAG (Gardner *et al.*, 1998b), and the latter forms a reactive quinone imine but is not approved for clinical use due to concerns of potential IDR cases during development (Lai *et al.*, 2000). One hypothesis to explain the increased IDIAG risk for clozapine compared with olanzapine is dose, because the therapeutic dose of olanzapine for schizophrenic patients is around 10–15 mg/day (Latifeh *et al.*, 2019), compared with typical clozapine regimens of up to 600 mg/day (Subramanian *et al.*, 2017). It is unlikely that dose is the only factor as described previously, in our rat model, we found that an equimolar dose of olanzapine to clozapine did not trigger the same increase in peripheral blood neutrophils (Ng *et al.*, 2014). Likewise, in this work, using comparable concentrations of clozapine, olanzapine, and fluperlapine, only clozapine induced inflammasome activation *in vitro* or *in vivo*. For olanzapine, this supports the working paradigm that drugs not associated with IDR risk do not activate inflammasomes. This is an important observation, because the THP-1 cells employed contain the necessary enzyme, myeloperoxidase, to bioactivate olanzapine to the reactive nitrenium ion that irreversibly binds proteins (Gardner *et al.*, 1998a). Other investigations have shown that the toxicity of the nitrenium ion of olanzapine is substantially less than clozapine (Gardner *et al.*, 1998a,b), underscoring the necessity of an additional signal, the release of DAMPs, for inflammasome activation to occur (Zheng *et al.*, 2020). If covalent binding of the reactive species does not evoke the release of DAMPs, then there will unlikely be sufficient stimuli to trigger inflammasome activation or induction of the host immune response. The detection of clozapine covalent binding to THP-1 macrophages and subsequent inflammasome activation support further investigation of this hypothesis.

It is unclear whether fluperlapine can cause IDAG. In contrast to clozapine, it cannot be directly bioactivated by THP-1 cells and must first be oxidized to 7-hydroxyfluperlapine, which can then be metabolized by myeloperoxidase to the reactive iminoquinone (Lai *et al.*, 2000). Thus, it is unsurprising that fluperlapine did not directly activate inflammasomes in our *in vitro* model. Fluperlapine, however, also did not induce a proinflammatory response in our rat model. It may be that fluperlapine does cause an innate response, but the onset occurs later (>24 h) or at a higher dose (ie, >28.4 mg/kg) than characterized here. What was similar between fluperlapine and clozapine *in vivo*, and has previously been observed with olanzapine (Ng *et al.*, 2014), was a decrease in lymphocytes, indicating that a second mechanism is contributing to leukocyte chemotaxis that, with clozapine, occurs concurrent to inflammasome-dependent neutrophil mobilization. Likewise, clozapine-induced neutrophil increases, but not lymphocyte decreases, were attenuated by VX-765 or anakinra, supporting the involvement of parallel chemotactic mechanism that appears to be conserved across structurally similar compounds. Due to the acute onset of lymphopenia, one probable cause is activation of endogenous corticosteroid signaling (Fauci, 1975; Marté *et al.*, 2020; Zweiman *et al.*, 1984). Corticosteroids can result in a profound yet transient decrease in peripheral lymphocytes within hours, similar to the pattern observed herein.

Several questions arise from the present work, including the specific inflammasome(s) responsible for initiation of clozapine-mediated inflammation. A role for NLRP3 seems plausible due to the extensive range of sterile stimuli detected by this inflammasome (He *et al.*, 2016), however, this remains to be investigated using inhibitors targeting specific inflammasome receptors, such as the clinically approved drugs Diacerein or Tranilast (Caseley *et al.*, 2020). Additionally, detection of inflammasome activation in patients is required, but low clozapine prescribing rates (Meltzer, 2012) and confounding antipsychotics cotreatments (Lam, 2021) represent significant challenges. Many psychiatric disorders have also been postulated to involve inflammation (Marazziti *et al.*, 2018; Miller and Raison, 2016; Rodrigues-Amorim *et al.*, 2018) and increased inflammasome subunit levels have been reported with schizophrenia spectrum disorders (Hylén *et al.*, 2020; Kim *et al.*, 2016), further complicating the delineation of clozapine-induced inflammasome activation in patients. Here, cotreatment with anakinra posed a challenge due to the disparity in results obtained with different lots, highlighting the concern of batch-to-batch variability inherent to biologics (U.S. Food and Drug Administration, 2013). Even with controlled manufacturing processes, small variations in biologic properties can arise, making it virtually impossible to produce identical batches (Patel *et al.*, 2015). Therefore, the use of VX-765 was deemed more appropriate that anakinra.

Rigorous hematological monitoring has reduced the mortality of IDIAG, with recent estimates at 5% (Pick and Nystrom, 2014), down from 20% several decades ago (Curtis, 2017). Despite these improvements, no diagnostic tests can reliably predict which clozapine patients will develop agranulocytosis (Verbelen *et al.*, 2015). Although several HLA associations have been identified, screening assays failed to yield adequate sensitivity in testing (Mijovic and MacCabe, 2020), and the risk of agranulocytosis precludes the routine use of clozapine. Inhibition of inflammasome activation during the initiation of clozapine treatment represents a potential strategy to circumvent the need for other diagnostic criteria. Without the development of an inflammatory response to clozapine, progression to an adaptive immune response that may culminate in IDIAG is unlikely. Although this approach has not been clinically tested, VX-765 has been demonstrated to have acceptable safety profiles during clinical trials for epilepsy (Vertex Pharmaceuticals Inc., 2014) and psoriasis (Vertex Pharmaceuticals Inc., 2007), representing a potential future direction for prevention of IDIAG onset.

A better mechanistic understanding of the immune response to clozapine, as demonstrated in this article, may help predict and prevent progression to IDIAG, enabling safer, more frequent use of clozapine to manage schizophrenia. Many of the characteristics of other IDRs are similar, and inflammasome activation may represent a conserved mechanism by which drugs trigger an inflammatory response that precedes IDR onset. Circumstantial evidence indicates that many IDRs are caused by reactive metabolites, yet many drugs that form reactive metabolites are not associated with a significant risk of IDRs. It is likely that, unless a reactive metabolite can induce an innate immune response, it will be unable to produce an adaptive immune response that, in some patients, leads to an IDR. Ultimately, elucidating the early steps in the induction of an immune response to drugs could be used to screen drug candidates for the potential to cause serious IDRs, facilitating the development of safer drugs.
